# Effects of an 8-week multimodal program on thoracic posture, glenohumeral range of motion and serve performance in competitive young tennis players

**DOI:** 10.3389/fspor.2023.1128075

**Published:** 2023-03-03

**Authors:** Tom Le Solliec, Yoann Blache, Isabelle Rogowski

**Affiliations:** Unité de Formation et de Recherche en Activités Physiques et Sportives, Université de Lyon, Université Lyon 1, Laboratoire Interuniversitaire de Biologie de la Motricité—EA 7424, UFRSTAPS, Villeurbanne Cedex, France

**Keywords:** overhead sport, self-myofascial release, stretching, strengthening, breathing

## Abstract

**Introduction:**

Intensive tennis practice is known to generate sport-specific adaptations at the shoulder region and influence the sagittal spinal curvature. However, increased thoracic kyphosis decreases the shoulder functional capacity, which could limit tennis performance. Therefore, the aim of this study was to investigate the effects of an 8-week multimodal program on thoracic posture, glenohumeral range of motion, and serve performance in competitive young tennis players.

**Methods:**

Eighteen male and four female players (age: 16.0 ± 2.4 years, height: 170.7 ± 11.0 cm; mass: 62.1 ± 11.5 kg; International Tennis Number: 3–4) performed their regular training during 8 weeks, which was used as a reference period, and implemented a multimodal program including stretching, strengthening, and myofascial release exercises, four times per week during 8 additional weeks, which corresponded to the intervention period. The thoracic curvature angle and mobility, the biacromial and interscapular distances, the glenohumeral range of motion and the tennis serve performance were assessed three times, i.e., before and after the regular training and after the 8-week multimodal program.

**Results:**

The results showed that the 8-week regular training had no significant effects on thoracic curvature angle [effect size (ES) = 0.02–0.36, *p* = 0.06–0.46] and mobility (ES = 0.05–0.26, *p* = 0.13–0.42), biacromial (ES = 0.05, *p* = 0.18) and interscapular distances (ES = 0.03, *p* = 0.45), ranges of motion in glenohumeral internal (ES = 0.04, *p* = 0.43) and external rotation (ES = 0.43, *p* = 0.06), and tennis serve accuracy (ES = 0.33, *p* = 0.07) and velocity (ES = 0.09, *p* = 0.35). The 8-week multimodal program increased moderately the thoracic mobility (ES = 0.55, *p* = 0.01), moderately to strongly the serve accuracy and velocity (ES = 0.65, *p* = 0.003, for both), strongly decreased the interscapular distance (ES = 1.02, *p* < 0.001), and strongly increased the range of motion in glenohumeral internal (ES = 0.90, *p* < 0.001) and external rotation (ES = 1.49, *p* < 0.001).

**Discussion:**

These findings indicated that an 8-week multimodal program, including spine and glenohumeral mobility and shoulder girdle strength exercises, performed four times per week during 8 weeks, is moderately relevant to rectify the sagittal thoracic curvature in competitive tennis players, while such a program may help regain the range of motion in glenohumeral rotation without tennis serve performance impairment.

## 1. Introduction

The achievement of tennis stroke is based on the kinetic chain concept (1), involving a sequential force development from the legs and trunk funneled through the shoulder complex and transferred to the upper extremity up to the racket to impact the ball with maximal velocity (2). The repetitive forceful unilateral movements lead to tennis-specific adaptations, especially at the shoulder region, such as decreased glenohumeral range of motion (3), imbalance in glenohumeral rotator muscle strength (4), or alterations in scapular positioning and motion (5). These adaptations can create disruption in the kinetic chain, possibly resulting in altered performance (1) or constituting risk factors for overuse injury, especially at the shoulder region (6). Because it must provide an efficient linkage to transfer forces from proximal to distal segments, the shoulder complex must benefit from a particular emphasis in the prevention program for tennis players.

The tennis serve is a key stroke to take advantage over an opponent during match (7). Due to the overhead arm motion, the dominant arm adopts extreme positions (8), known to increase the contact pressure and area of impingement of the rotator cuff tendon between the humeral head and glenoid cavity (9, 10). In particular, at the end of the cocking phase, the humerus is in maximal external rotation, abduction, and extension (11), and, at impact, the humerus elevates at about 100° (12) in the frontal plane (11). The achievement of these extreme positions demands coordinated motions of the humerus and the scapula (11), on the one hand, and contribution of the spine (13), on the other hand. Indeed, the trunk extension contributes to the scapular posterior tilt, which contributes itself to the humeral external rotation (14), and to the arm elevation (15). In addition, repetitive powerful overhead movements lead to imbalance in length and strength between anterior and posterior shoulder muscles, fostering forward head and shoulder posture (16, 17). Repetitive trunk forward-bending and extension also influence spinal profile of tennis players (18), in particular, increased thoracic kyphosis (19). A combination of forward head posture, forward shoulder posture, and increased thoracic kyphosis is described as slouched posture, which impairs the shoulder functions. Such a slouched posture angle is associated with decreased range of motion in glenohumeral external rotation (20, 21) and arm abduction (20, 22), decreased glenohumeral external rotator muscle strength, and decreased scapular posterior tilt (20, 23). Like positive correlations have been reported between tennis serve velocity and glenohumeral range of motion and strength (24), preventing the consequences of deficiencies in trunk extension and acquired slouched posture on shoulder functions involved to achieve tennis stroke may be a goal of prevention program for tennis players.

To preserve tennis players' shoulder functions, previous studies (25–28) have mainly focused on the glenohumeral joint to independently prevent the decrease in internal rotation range of motion (IROM) and the imbalance in strength of external and internal rotator muscles. The glenohumeral internal rotation range of motion may be preserved or increased when performing either stretching exercises (25) or self-myofascial release (28). Rebalancing the strength of glenohumeral external rotators in regard with strength of internal rotators may be achieved by isokinetic training of eccentric external rotator muscle strength (26) or by sling-based exercise for external rotator muscles (27). To the best of our knowledge, no study investigates the effects of trunk exercise on the spinal curvature in tennis players. However, in swimmers, respiratory muscle exercise by stimulating the local trunk stabilizers straightens the thoracic spine (29). A recent meta-analysis highlights that intervention programs including both strengthening and stretching exercises have large statistically significant effects for reducing the curve of thoracic angle (30). Consequently, a prevention program including strengthening and stretching of the upper trunk and glenohumeral joint may be interesting to maintain thoracic alignment and mobility and to preserve shoulder functions in tennis players.

Therefore, the aim of this study was to investigate the effects of an 8-week multimodal program on thoracic posture, glenohumeral range of motion, and serve performance in competitive young tennis players. It was hypothesized that this program would straighten the thoracic spine, increase glenohumeral rotational range of motion, and improve tennis serve performance.

## 2. Methods

### 2.1. Participants

A sample size calculation was performed using a large effect size, based on the results of the meta-analysis reported by Gonzalez-Galvez et al. (30). The *a priori* statistical power analysis indicated a sample size of a minimum of 18 participants assessed three times, with *α* = 0.05, statistical power = 0.95, and effect size f = 0.40. Given the duration of the study (16 weeks), we expected a risk of 20% of players lost to follow-up.

Eighteen male and four female tennis players [age: 16.0 ± 2.4 years, height: 170.7 ± 11.0 cm; mass: 62.1 ± 11.5 kg; predicted age at peak height velocity (31): 1.4 ± 1.9 years; weekly tennis training: 7.6 ± 2.4 h; weekly strength and conditioning training: 4.2 ± 0.4 h; tennis experience: 9.8 ± 2.2 years; International Tennis Number: 3–4, advanced players] volunteered to participate in this study, which was approved by the local ethics committee. All participants were recruited from a tennis academy. Inclusion criteria were being aged between 13 and 25 years, playing competitive tennis, and training at least four times a week. Exclusion criteria were having pain during tennis playing or injury (defined as problems resulting in tennis playing time loss higher than 2 weeks), history of surgery at the dominant upper limb or trunk within the previous 6 months, or having significant postural alterations, such as scoliosis or hyperkyphosis.

### 2.2. Study design

A test–retest procedure was applied over a 16-week duration, during which the training workload remained similar. The players were assessed at baseline (T_0_), after 8 weeks (T_1_), and after 16 weeks (T_2_). During the control period between T_0_ and T_1_, i.e., from November to January, the players performed their regular training. During the intervention period between T_1_ and T_2_, i.e., from January to March, the players implemented the multimodal program four times per week at the beginning of their strength and conditioning sessions.

### 2.3. Testing procedures

The demographic and tennis characteristics were collected at baseline (T_0_). The thoracic curvature, biacromial and interscapular distances, glenohumeral ranges of motion, and serve performance were assessed by the same examiners at T_0_, T_1_, and T_2_.

#### 2.3.1. Thoracic spine curvature

**Figure 1:**
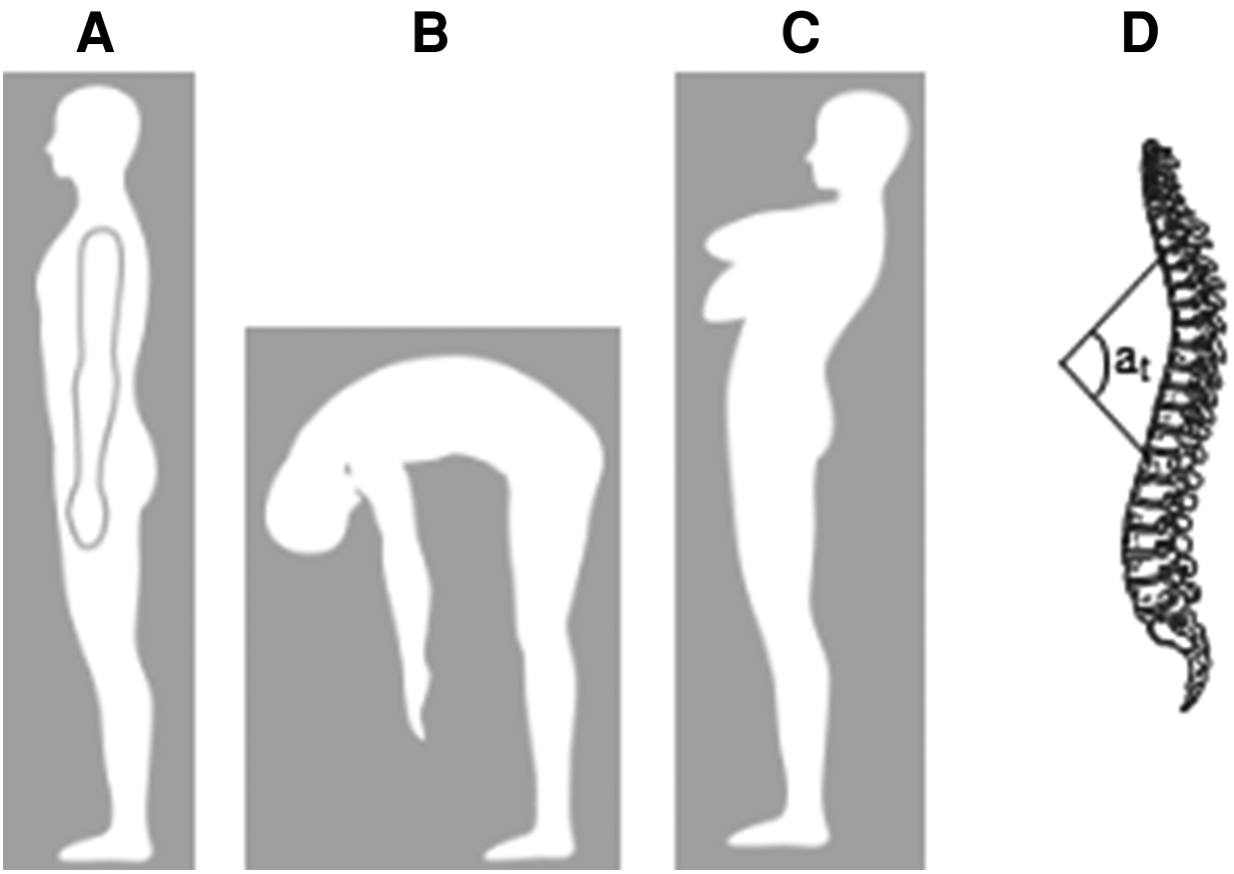
Sagittal spinal curvatures assessment in three trunk positions. (**A**) neutral standing, (**B**) maximal trunk flexion, (**C**) maximal trunk extension, and (**D**) thoracic angle a_t_.

Sagittal spinal curvatures were assessed in three trunk positions successively: neutral standing (Figure 1A), maximal trunk flexion with stretched legs (Figure 1B), and maximal trunk extension (Figure 1C), using the Spinal Mouse system (IDIAG-M360pro, Fehraltorf, Suisse), which provides reliable measurements of thoracic curvatures and range of motion in the sagittal profile (32). The examiner marked the spinous processes of the seventh cervical (C7) and third sacral (S3) vertebrae, then put the Spinal Mouse on C7, and guided it along the midline of the spine to S3. The thoracic spine angle (Figure 1D) was measured in each trunk position, and thoracic spine mobility was evaluated by differences in thoracic angles between paired trunk positions, i.e., between neutral standing and flexion positions, between neutral standing and extension positions, and between extension and flexion positions. No reliability assessments were made for these outcome measures.

#### 2.3.2. Biacromial distance

The biacromial distance was measured using a spreading caliper. While the player is in a neutral standing position, the examiner puts the ends of the caliper on the acromion anterior part of each shoulder to measure the biacromial distance [the intrasession reliability for the examiner performing the measurements was as follows: intraclass coefficient correlation (ICC) = 0.97; standard error of measurement (SEM) = 0.37 cm; minimal detectable change at 95% confidence level (MDC_95%_) = 1.03 cm]. The distance was measured twice and averaged for subsequent analysis. Short biacromial distance reflects the forward shoulder posture.

#### 2.3.3. Interscapular distance

The interscapular distance was measured using a spreading caliper. While the player was in a neutral standing position, the examiner puts the ends of the caliper on the inferior part of medial border of each scapula to measure the interscapular distance (intrasession reliability: ICC = 0.97; SEM = 0.35 cm; MDC_95%_ = 0.98 cm). The distance was measured twice and averaged for subsequent analysis.

#### 2.3.4. Glenohumeral range of motion

The range of motion at the dominant glenohumeral joint was assessed in internal (IROM) and external rotation (EROM) using using a bubble goniometer in accordance with the previously described procedure (33). The player was in a supine position with the humerus abducted at 90° and elbow flexed at 90°. An examiner maintained the coracoid process and scapular spinae, and then internally or externally rotated the upper arm to the maximum until just before the first motion of the scapula. Another examiner located the goniometer center on the olecranon process with the bubble-branch vertically and the other aligned onto the forearm to measure IROM (intrasession reliability: ICC = 0.98; SEM = 1.1°; MDC_95%_ = 3.5°) and EROM (intrasession reliability: ICC = 0.99; SEM = 0.9°; MDC_95%_ = 2.4°). The measurements were performed twice for each rotation, and averaged for subsequent analysis. Mean IROM and mean EROM were summed to compute the total arc of motion (TAM).

#### 2.3.5. Serve performance

The serve performance was assessed by the serve accuracy and ball velocity. After a general warm-up composed of arm internal/external rotation, arm and elbow flexion/extension using elastic band, and forehand and backhand strokes, the players performed a specific warm-up composed of eight serves at 50% of maximal effort, four serves at 75%, and four serves at 90%. Then, each player was instructed to perform 12 first serves, 6 serves per diagonal, as fast as possible, while looking for ace on the “T” of the serve box. The serve accuracy was evaluated using a point system (34). Briefly, two targets of 50 cm × 50 cm and 1 m × 1 m were placed from the middle line to the serve line of the service box. A rebound in the small target accounted for 5 points, in the big target for 3 points, in another location in the serve box for 1 point, and another location and in the net for 0 point (please see Supplementary Figure 1). The points obtained for the 12 serves were summed to compute the score for serve accuracy. The rate of successful serves (i.e., rebound in the opposite serve box) was also computed. The ball velocity was measured using a radar gun (Stalker Pro II, Stalker Radar, Plano, TX, United States), located 2.50 m behind the player at 1.7 m height. The maximal velocity obtained for a successful serve, i.e., ball rebound in the serve box, was kept for the subsequent analysis.

### 2.4. Intervention program

Players and coaches were blinded for the purpose of the study, instructed to bring no technical changes in the player's serve, while maintaining the duration of the tennis training at the same level throughout the 16-week procedure. The regular tennis training consisted of general and targeted warm-up, exercises to control ball direction and depth in basic strokes, tactical games, and training matches (35). During the control period, the players performed their usual prevention protocol at the beginning of the strength and conditioning session, such as one pectoralis stretching exercise (4 min) followed by three series of 10 YWTL movements executed without additional load (8 min) to strengthen posterior upper trunk muscles. Y, W, T, and L describe the position of the upper extremities relative to the thorax (please see figures in Supplementary Table 1). During the intervention period, the usual prevention protocol was replaced with the multimodal program for the same duration, including three self-myofascial release exercises (2.5 min) onto anterior upper trunk and posterior shoulder areas, three stretching exercises (2.5 min) of the anterior and posterior shoulder structures, three trunk mobility exercises combined with breathing instructions (3 min), and six strengthening exercises (4 min) targeting posterior upper trunk muscles. All the sessions of both the control and intervention periods for all the players were supervised by the same strength and conditioning coaches. All the exercises are fully described in Supplementary Tables 1 and 2.

### 2.5. Statistical analyses

All data are presented as mean ± SD. After checking the normality and homoscedasticity of the raw data with the Shapiro–Wilk and Levene tests, respectively, ANOVAs for three repeated measures (Time: T_0_ vs. T_1_ vs. T_2_) were applied to evaluate the effect of time on thoracic spine angles and mobility, shoulder girdle distances, glenohumeral ranges of motion, and serve performance with reporting partial effect sizes (f; 0.10 for small effect, 0.25 for medium effect, and 0.40 for large effect) and *p*-value. When ANOVA revealed a significant effect of time, Bonferroni-corrected *post-hoc test*s were applied to compare the results between T_0_ and T_1_, i.e., the changes related to the control period, and between T_1_ and T_2_, i.e., the changes in relation with the intervention period with reporting effect size [effect size (ES): 0.2 for small effect, 0.5 for medium effect, and 0.8 for large effect] and *p*-value. For all the statistical tests, Rcmdr package of the software R 4.1.0 (R, Foundation for Statistical Computing, Vienna, Austria) was used, and the level of significance was set at *p* ≤ 0.05.

## 3. Results

All competitive young tennis players included in this study performed all the program sessions in both the control and intervention periods. None of them sustained injury demanding to be excluded from the study.

For the thoracic spine angles (Table 1), ANOVA revealed no significant effect of time in the neutral standing position (f = 0.09, low effect; *p* = 0.06), while a significant effect of time was found in flexion (f = 0.20, low-to-medium effect; *p* = 0.005) and extension (f = 0.11, low effect; *p* = 0.05) positions. In the flexion position, no significant changes were found either after the control period or after the intervention period (the significant effect between T_0_ and T_2_ was out of interest for our purpose). In the extension position, the mean thoracic spine angles were similar between T_0_ and T_1_ (ES = 0.02, low effect; *p* = 0.46) and decreased significantly between T1 and T2 (ES = 0.40, medium effect; *p* = 0.04). The changes in thoracic curvature angles are presented in Supplementary Figure 2.

**Table 1:** Mean (±SD) postural outcome measures at baseline (T_0_), after the control period (T_1_). and after the intervention period (T_2_).

	Position	T_0_	T_1_	T_2_	
Thoracic spine angle (°)	Neutral standing	37.5 ± 9.9	40.2 ± 9.3	37.4 ± 7.4	
	Flexion	48.6 ± 12.9	52.8 ± 10.9	56.3 ± 12.1	
	Extension	34.6 ± 15.3	34.4 ± 12.7	29.3 ± 12.4	*
Thoracic spine mobility (°)	Neutral standing–flexion	12.0 ± 10.8	12.6 ± 11.8	18.9 ± 13.3	**
	Neutral standing–extension	−2.5 ± 14.4	−5.8 ± 12.2	−8.1 ± 11.4	
	Extension–flexion	14.4 ± 20.8	18.4 ± 16.4	27.0 ± 15.1	**
Biacromial distance (cm)		32.9 ± 2.2	33.0 ± 2.3	33.3 ± 2.9	
Interscapular distance (cm)		15.4 ± 1.9	15.3 ± 1.5	14.3 ± 1.6	***

*Significant difference between T_1_ and T_2_, with for p ≤ 0.05; **Significant difference between T_1_ and T_2_, with for p ≤ 0.01; ***Significant difference between T_1_ and T_2_, with for p ≤ 0.001.

For the thoracic spine mobility (Table 1), no significant effect of time was found when assessed between neutral standing and extension positions (f = 0.06, low effect; *p* = 0.13), while a significant effect of time was found when mobility was measured between neutral standing and flexion positions (f = 0.17, low-to-medium effect; *p* = 0.01), and extension and flexion positions (f = 0.20, low-to-medium effect; *p* = 0.005). For these last two mobilities, no changes were reported after the control period (ES = 0.05, low effect; *p* = 0.41, and ES = 0.21, low effect; *p* = 0.16, respectively), while significant increases were observed after the intervention period (ES = 0.53, medium effect; *p* = 0.01 and ES = 0.55, medium effect; *p* = 0.009, respectively).

For the shoulder girdle distances (Table 1), no effect of time was observed on the biacromial distance (f = 0.04, low effect; *p* = 0.18). ANOVA revealed a significant effect of time on the interscapular distance (f = 0.42, large effect; *p* < 0.001), which were similar at T_0_ and T_1_ (ES = 0.03, low effect; *p* = 0.45), and significantly lower at T_2_ compared to T1 (ES = 1.02, large effect; *p* < 0.001).

For the glenohumeral ranges of motion (Table 2), ANOVA revealed a significant effect of time for IROM (f = 0.36, medium-to-large effect; *p* < 0.001), EROM (f = 0.55, large effect; *p* < 0.001), and TAM (f = 0.59, large effect; *p* < 0.001). IROM, EROM, and TAM were similar at T_0_ and T_1_ (ES = 0.04, low effect; *p* = 0.43; ES = 0.33, low effect; *p* = 0.06; and ES = 0.34, low effect; *p* = 0.06, respectively), and increased significantly between T_1_ and T_2_ (ES = 0.90, large effect; *p* < 0.001; ES = 1.49; large effect; *p* < 0.001; and ES = 1.52, large effect; *p* < 0.001, respectively).

**Table 2:** Mean (±SD) ranges of motion (in °) in IROM and EROM rotation and TAM at dominant glenohumeral joint at baseline (T_0_), after the control period (T_1_), and after the intervention period (T_2_).

	T_0_	T_1_	T_2_	
IROM	39.0 ± 8.1	39.3 ± 8.3	46.7 ± 5.4	***
EROM	87.0 ± 13.1	91.0 ± 10.1	100.0 ± 7.6	***
TAM	126.0 ± 15.4	130.3 ± 13.8	146.7 ± 7.4	***

IROM, internal rotation range of motion; EROM, external rotation range of motion; TAM, total arc of motion.

***Significant difference between T_1_ and T_2_, with for p ≤ 0.001.

Regarding the serve performance (Table 3), ANOVA revealed a significant effect of time on accuracy (f = 0.35, medium-to-large effect; *p* < 0.001), rate of successful serves (f = 0.23, medium-to-large effect; *p* = 0.002), and velocity (f = 0.14, low-to-medium effect; *p* = 0.02). The mean accuracy, rate of successful serves, and velocity remained similar between T_0_ and T_1_ (ES = 0.33; low effect; *p* = 0.07; ES = 0.03; low effect; *p* = 0.44; and ES = 0.07; low effect; *p* = 0.34, respectively), and all increased significantly between T_1_ and T_2_ (ES = 0.65; medium-to-large effect; *p* = 0.003; ES = 0.68; medium-to-large effect; *p* = 0.003; and ES = 0.54; medium effect; *p* = 0.003, respectively).

**Table 3:** Mean (±SD) tennis serve performance at baseline (T_0_), after the control period (T_1_), and after the intervention period (T_2_).

	T_0_	T_1_	T_2_	
Accuracy (points)	10.8 ± 6.1	13.1 ± 7.3	18.1 ± 6.1	**
Successful serves (%)	41 ± 19	41 ± 16	55 ± 13	**
Velocity (km h^−1^)	163.3 ± 16.8	162.7 ± 16.9	166.2 ± 16.5	**

**Significant difference between T_1_ and T_2_, with for p ≤ 0.01.

## 4. Discussion

This study aimed to investigate the effects of an 8-week multimodal program on thoracic posture, glenohumeral range of motion, and serve performance in competitive young tennis players. The main findings were that the 8-week multimodal program erected the thoracic posture in trunk extension position, improved thoracic mobility, increased scapular medially rotated position, increased internal and external rotation range of motion at the dominant glenohumeral joint, and improved tennis serve performance.

Long-term exposure to tennis practice leads to morphological (36), muscular (4), and bony adaptations (37) enlarged when practice begins at young age (38). Our players with an average experience about 10 years of tennis practice presented with upper trunk and shoulder adaptations commonly observed with cumulative overhead activity exposure in overhead sport athletes (39) and tennis players (18, 19). Mean thoracic curvature angles in the neutral standing position for our players were near the upper values for normal thoracic kyphosis, i.e., between 20° and 45° (40), and remained close to those reported in tennis players of similar ages (19). Regarding glenohumeral flexibility, the limitation in internal rotation range of motion at the dominant side is not fully compensated by the increase in external rotation range of motion, leading to decreased total arc of motion (3, 33). The tennis players involved in this study presented with similar ranges of motion in internal and external rotation than previously reported for tennis players of similar age and level (3) (Le Gal, 2018), and lower than those reported for controls (41). Currently, there is a consensus on the relationship between reduced glenohumeral range of motion, glenohumeral strength unbalance, and increased risk of shoulder injury (6); therefore, regaining the range of motion and strength balance at the dominant glenohumeral joint may be an integral part of any prevention strategy to recover shoulder functions in overhead athletes (42). Although our tennis players performed their regular tennis training and prevention program composed of one stretching exercise and the four YWTL exercises without load, no changes in either trunk posture (18) or glenohumeral flexibility (Le Gal, 2018) were observed after the 8-week control period. Consequently, implementing exercises targeting stretching, strengthening, and mobility of the upper trunk and shoulder region may help counteract the effects of long-term tennis practice on upper trunk curvature angle and shoulder range of motion to preserve tennis performance and preserve shoulder functions.

The achievement of tennis strokes, especially tennis serve, applies asymmetric loads, creating the progression of muscular asymmetry and spine alterations (18). Particularly, movements combining trunk flexion and hyperextension motions result in shortening the anterior muscles and lengthening the posterior muscles, leading to increased forward head and shoulder posture, and thoracic kyphosis (16). When implementing four times per week during 8 weeks, stretching exercises for anterior trunk and shoulder muscles, strengthening exercises with load for posterior muscles, and mobility exercises while performing deep breathing resulted in medium statistically significant changes in thoracic alignment in extended body position and thoracic mobility. Although the design of our multimodal program respected the content, frequency, and duration recommended by the systematic review of Gonzalez-Galvez et al. (30), the changes in thoracic alignment obtained in our tennis players remained lesser than expected. Such discrepancies may be explained by differences in terms of participant characteristics, initial thoracic posture, or nature of the study. However, small changes in thoracic posture have immediate effect on shoulder range of motion (43). An erect thoracic posture is known to increase scapular posterior tilt (20), scapular upward rotation (14), and scapular medial rotation (14); such scapular positioning contributes to reaching a more extreme external rotated position of the arm when it is abducted, as at the end of the cocking phase of the tennis serve (13). Interestingly, our multimodal program also resulted in a more medially rotated position of the scapulae, which, concomitant with thoracic realignment in extension trunk posture, may possibly enlarge the subacromial space during arm elevation (43). Additionally, the stretching exercises and self-myofascial releases of the dominant shoulder region included in our multimodal program lead to an increase close to 20% in glenohumeral internal rotation range of motion, which was in accordance with previous reported gains (28). It may be hypothesized that the benefits in upper trunk posture associated with this increase in dominant glenohumeral range of motion may contribute to the increase in the range of motion of the cocking phase and then the course of the acceleration phase of the tennis serve, which may explain the gain in serve velocity. Moreover, it may be also supposed that the gain in thoracic, scapular, and glenohumeral movements may improve the intersegmental positioning of the upper limb to increase the accuracy of the tennis serve. Our findings thus indicated that a multimodal program focusing on upper trunk and shoulder including stretching, strengthening, mobility, and respiratory exercises performed four times per week during 8 weeks was not effective enough to alter the thoracic postural curvature in the standing position, but programming such a program was relevant to increase spine and shoulder mobility and improve tennis serve performance.

This study presents limitations that warrant discussion. First, strength assessments were not performed neither for glenohumeral muscles nor for scapular muscles, not allowing the effects of strength exercises to be related to scapular positioning. Second, a tennis serve kinematic analysis may help evaluate whether a transfer of the decreased thoracic curvature during trunk extension occurred during the tennis serve motion. Third, the effects of the multimodal program on postural and shoulder adaptations may be influenced by the large range in tennis experience due to age dispersion of our players and/or by the natural evolution of the tennis practice when control and intervention periods were performed successively in different periods of the tennis season. This study was, however, the first investigating the effects of a multimodal program acting on both the spine and shoulder girdle region in competitive young tennis players. Further studies need to evaluate whether such a multimodal program may have effects on the forward posture in the long term, as well as to better understand the transfer of such multimodal program effects into the sport-specific performance.

## 5. Practical applications

A multimodal program focusing on upper trunk and shoulder including stretching, strengthening, mobility, and respiratory exercises performed four times per week during 8 weeks has no effect on the thoracic spine alignment; but it is relevant to gain in thoracic, scapular, and glenohumeral mobility in competitive young tennis players. Such a program could be implemented regardless of the season periodization because its gains are transferred to the tennis-specific performance.

## 6. Conclusion

The findings of the present study showed that a multimodal program including spine and glenohumeral mobility and shoulder girdle strength exercises resulted in improved thoracic mobility, decreased interscapular distance, increased glenohumeral rotation range of motion, and increased tennis serve performance. This study brings new knowledge to tennis and strength and conditioning coaches, and clinicians to prevent shoulder function limitations without altering sport performance in overhead athletes.

## Data Availability

The raw data supporting the conclusions of this article will be made available by the authors, without undue reservation.
