# Supplementary material for: Effects of an 8-week multimodal program on thoracic posture, glenohumeral range of motion and serve performance in competitive young tennis players
Source: Front Sports Act Living. 2023 Mar 3;5:1128075. doi: 10.3389/fspor.2023.1128075 (PMC10020231; doi:10.3389/fspor.2023.1128075)
Supplement: Supplementary file 1 [file Datasheet1.pdf]

Supplementary Material

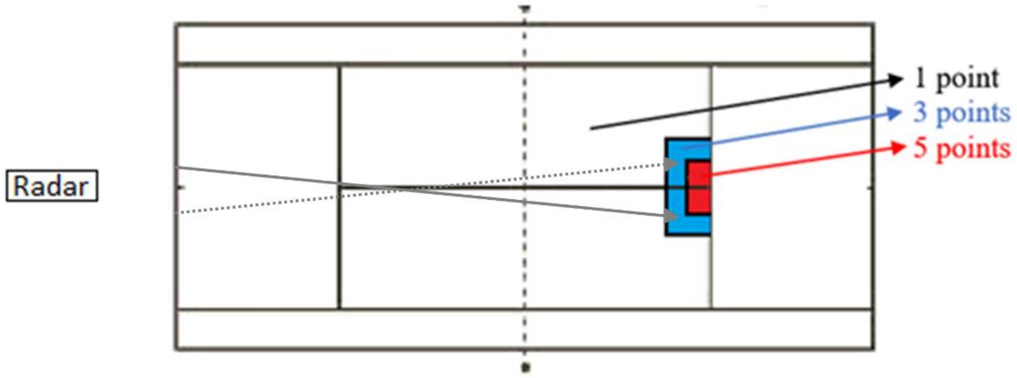

Supplementary Figure 1. Testing tennis court instrumentation.

The grey arrow represents the serve ball trajectory on the advantage diagonal, while the grey dot arrow, the serve ball trajectory on the deuce diagonal. For the scoring system, the ball bound in the serve box accounts for one point, in the blue target, for three points and in the red for five points.

PREVENTION PROGRAMS

Supplementary Table 1: Regular prevention program performed during the control period.

	Exercises	Illustration	Set	Reps	Load	Rest (s)	Starting position	Instructions
STRETCHING	Stretching: anterior muscle chain (Ruivo et al., 2017)	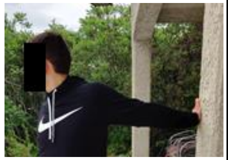	3	2	15’’	10	- Standing facing a wall<br>- Put one hand on the wall<br>- Keep your feet parallel	- Extend your arm<br>- Put one hand in supination<br>- Turn almost back to the wall to stretch the anterior muscle of the shoulder
STRENGTHENING	Strengthening of the posterior chain: Y (Ruivo et al., 2017)	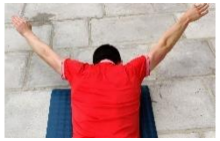	3	10	0	15	- Lie down on stomach<br>- Straight head<br>- Extend arms in abduction above the head	- Raise the arm by putting the scapula in adduction<br>- Keep shoulders down
	Strengthening of the posterior chain: W (Ruivo et al., 2017)	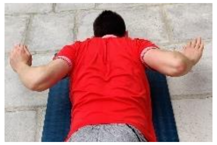	3	10	0	15	- Lie down on stomach<br>- Straight head<br>- Elbows bent	- Raise the arm by putting the scapula in adduction<br>- Keep shoulders down and elbows flex
	Strengthening of the posterior chain: T (Ruivo et al., 2017)	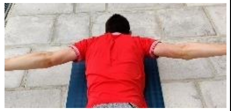	3	10	0	15	- Lie down on stomach<br>- Straight head<br>- Arms extended in 90 ° abduction	- Raise the arm by putting the scapula in adduction<br>- Keep shoulders down
	Strengthening of the posterior chain: L (Ruivo et al., 2017)	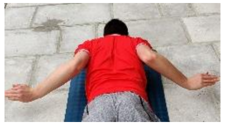	3	10	0	15	- Lie down on stomach<br>- Straight head<br>- Arms along the body and elbows bent	- Flex the elbows and put the scapula in adduction<br>- Perform an glenohumeral external rotation<br>- Keep shoulders down

Table 2: Prevention protocol including self-myofascial release, stretching, mobility, strengthening exercises performed during the intervention period.

	Exercises	Illustration	Set	Reps	Time (s)	Rest (s)	Starting position	Instructions
FLEXIBILITY	Self-myofascial release: anterior muscle chain (Le Gal et al., 2018)	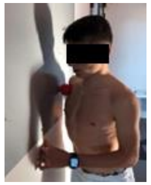	1	2	15	10	- Standing facing a wall<br>- Slightly three quarter<br>- Straight head	- Put the firm ball on the anterior part of the shoulder.<br>- Move along the wall applying pressure
	Stretching: anterior muscle chain (Ruivo et al., 2017)	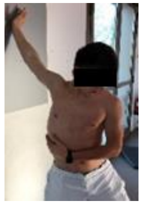	1	2	15	10	- Standing facing a wall<br>- Put one hand on the wall<br>- Keep your feet parallel	- Extend your arm<br>- Put one hand in supination<br>- Turn almost back to the wall to stretch the anterior muscle of the shoulder
	Self-myofascial release: posterior muscle chain (Le Gal et al., 2018)	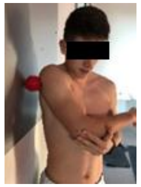	1	2	15	10	- Standing back to the wall<br>- Straight head	- Put the firm ball on the posterior part of the shoulder<br>- Move along the wall applying pressure
	Sleeper stretch (Kibler et al., 2012)	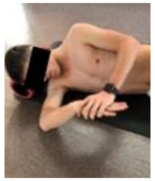	1	2	15	10	- Lie down on side<br>- Put a roller under the head<br>- Put one arm at 90 ° abduction on the floor<br>- Bend the elbow (90°)	- Perform manual stretching by applying downward pressure at the wrist
	Self-myofascial release: Latissimus dorsi (Monteiro et al., 2017)	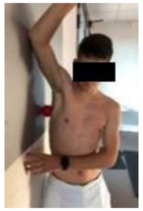	1	2	15	10	- Standing next to the wall<br>- Raise one arm<br>- Straight head	- Put the firm ball on the latissimus dorsi<br>- Move along the wall applying pressure

	Stretching: Latissimus dorsi (Turgut et al., 2018)	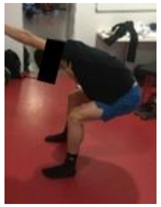	1	2	15	10	- Catch the TRX<br>- Keep your feet parallel and spread	- Pull the TRX and do a hip, knee and ankle flexion
MOBILITY	Spine flexion and extension (Sakata et al., 2019)	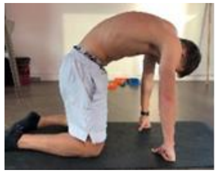 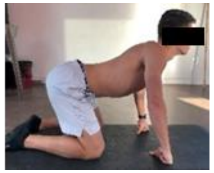	1	10	0	10	- Be in push-up position with knees on the floor	- Do a maximum flexion and extension of the entire spine<br>- Do a flexion and extension of the head<br>- At the same time perform several deep breaths
	Spine rotation (Sakata et al., 2019)	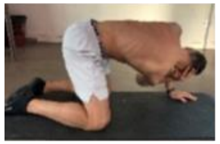 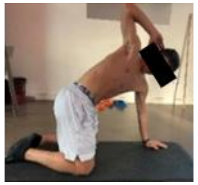	1	10	0	10	- Be in push-up position with knees on the floor<br>- One hand on the side of the head	- Perform a maximum spine rotation without hip movement<br>- Perform an adduction of the scapula at the end of the rotation
	Lying spine rotation (Leonard 1990)	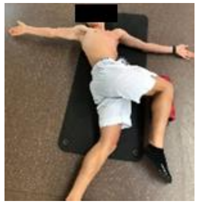	1	10	0	10	- Lie down on side<br>- One leg stretch and the other extend<br>- Put one roller under the stretched knee<br>- Extend arms on the side	- Perform a maximum spine rotation by opening the arms<br>- No hip movement
STRENGTHENING	Strengthening of the posterior chain: Y (Ruivo et al., 2017)	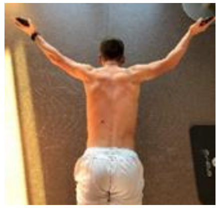	1	10	0.500kg<br>1kg<br>or 2kg<br>*	30	- Lie down on stomach (on a bench)<br>- Straight head<br>- Extend arms in abduction above the head	- Raise the arm by putting the scapula in adduction<br>- Keep shoulders down
	Strengthening of the posterior chain: W (Ruivo et al., 2017)	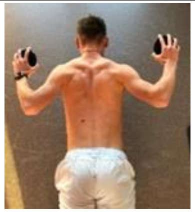	1	10	0.500kg<br>1kg<br>or 2kg<br>*	30	- Lie down on stomach (on a bench)<br>- Straight head<br>- Elbows bent	- Raise the arm by putting the scapula in adduction<br>- Keep shoulders down and elbows flex

	Strengthening of the posterior chain: T (Ruivo et al., 2017)	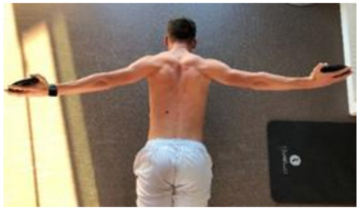	1	10	0.500kg 1kg or 2kg *	30	- Lie down on stomach (on a bench)<br>- Straight head<br>- Arms extended in 90 ° abduction	- Raise the arm by putting the scapula in adduction<br>- Keep shoulders down
	Strengthening of the posterior chain: L (Ruivo et al., 2017)	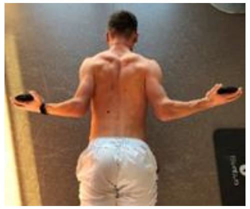	1	10	0.500kg 1kg or 2kg *	30	- Lie down on stomach (on a bench)<br>- Straight head<br>- Arms along the body and elbows bent	- Flex the elbows and put the scapula in adduction<br>- Perform an glenohumeral external rotation<br>- Keep shoulders down
	Scapular push-up (Genevois 2014)	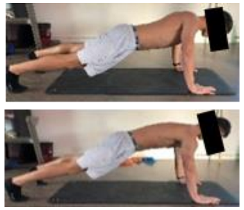	1	10	0	30	- In push-up position	- Perform a lateral rotation of the shoulder followed by adduction of scapula<br>- No spine movement
	Dead bug (McGill et al., 2009)	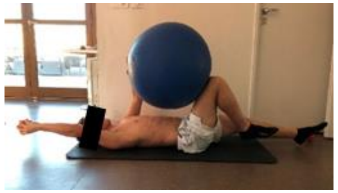	1	10	0	30	- Lie down on back<br>- 90° flexion of Hips and knees<br>- Put a swiss ball between hands and knees	- Extend one leg and raise the opposite arm<br>- Keep pressure on the swiss ball<br>- No lumbar hyper lordosis

*The demographic characteristics were used for the individualisation of the load of the protocol. Even if the duration of the protocol were similar for all players, each of them used a weight adapted to their level and their physical maturity. The youngest (PHV <1), used 500g weights to perform their strengthening exercises while the older (PHV> 1) used weights of 1kg (or more depending on their physical abilities).

References

Genevois C, Berthier P, Guidou V, Muller F, Thiebault B, Rogowski I. "Effects of 6-Week Sling-Based Training of the External-Rotator Muscles on the Shoulder Profile in Elite Female High School Handball Players". *Journal of Sport Rehabilitation.* 2014; 23:286-295.

Kibler BW, Sciascia A, Thomas SJ. "Glenohumeral Internal Rotation Deficit : Pathogenesis and Response to Acute Throwing". *Sports Medecine and Arthroscopy Review*. 2012; 20:34-38.

Le Gal J, Begon M, Gillet B, Rogowski I. "Effects of Self-Myofascial Release on Shoulder Function and Perception in Adolescent Tennis Players". *Journal of Sport Rehabilitation.* 2018; 27:530-535.

Leonard SA. "The Role of Exercise and Posture in Preventing Low Back Injury ». *American Association of Occupational Health Nurses Journal*. 1990; 38:318-22.

McGill SM, Karpowicz A. "Exercises for Spine Stabilization: Motion/Motor Patterns, Stability Progressions, and Clinical Technique". *Archives of Physical Medicine and Rehabilitation*. 2009; 90:118-26.

Monteiro ERJ, Vigotsky AD, Brown AM, Gomes TM. "Acute Effects of Different Self-Massage Tm Volumes On The Fms Overhead Deep Squat Performance". *International Journal of Sports Physical Therapy*. 2017; 12:94-104.

Ruivo RM, Pezarat-Correia P, Carita AI. "Effects of a Resistance and Stretching Training Program on Forward Head and Protracted Shoulder Posture in Adolescents". *Journal of Manipulative and Physiological Therapeutics.* 2017; 40:1-10.

Sakata J, Nakamura E, Suzuki T, Suzukawa M, Akeda M, Yamazaki T, Ellenbecker TS, Hirose N. "Throwing Injuries in Youth Baseball Players: Can a Prevention Program Help? A Randomized Controlled Trial". T*he American Journal of Sports Medicine*. 2017; 47:2709-16.

Turgut EID, Baltaci G. "Stretching Exercises for Subacromial Impingement Syndrome: Effects of 6-Week Program on Shoulder Tightness, Pain, and Disability Status". *Journal of Sport Rehabilitation*. 2018; 27:132-37

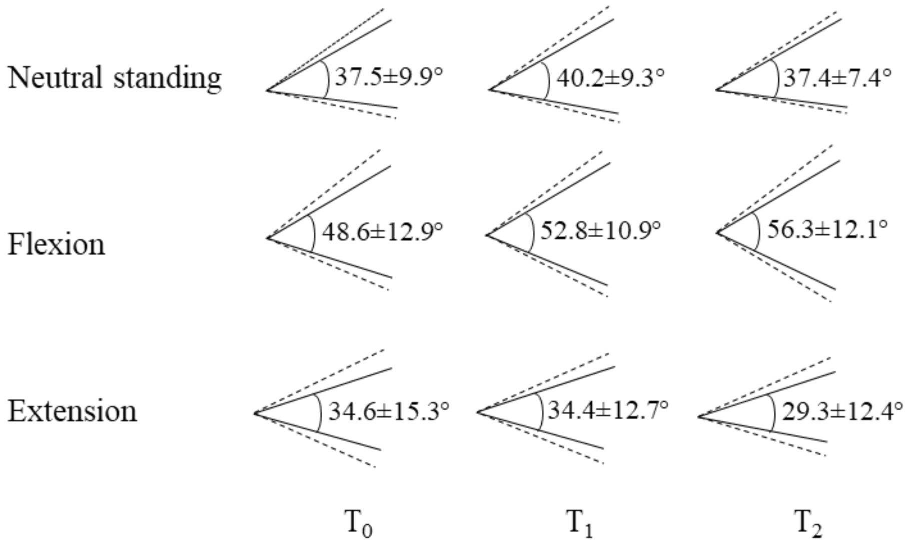

Supplementary Figure 2. Illustrations of the thoracic curvature angles (mean, in full line, + standard deviation, in dotted line) according to the body position (neutral standing, flexion, and extension) at baseline (T0), after the control period (T1) and after the intervention period (T2).
